# Animals as overlooked victims and vectors of tuberculosis: review of *Mycobacterium tuberculosis* and *Mycobacterium bovis*

**DOI:** 10.1186/s44280-026-00136-z

**Published:** 2026-07-10

**Authors:** Rachel C. Sadoff, Kunchok Dorjee

**Affiliations:** 1 Center for Tuberculosis and AIDS Research, Division of Infectious Diseases, Johns Hopkins University School of Medicine, Baltimore, MD 21287 USA; 2 Department of International Health, Johns Hopkins Bloomberg School of Public Health, Baltimore, MD 21205 USA

**Keywords:** Tuberculosis, One Health, Animals, Environment

## Abstract

Tuberculosis (TB) is the world′s leading infectious disease in humans usually caused by *Mycobacterium tuberculosis* (Mtb). However, *Mycobacterium bovis* can also lead to TB in humans and is the most common TB-causing bacteria in animals. Roughly 140,000 people fall sick from *M. bovis* every year, and tens of millions of animals are estimated to have TB infection, but there are major barriers to diagnosis and treatment in both humans and animals. For example, most standard TB tests do not detect *M. bovis*. *M. bovis* is also naturally resistant to pyrazinamide, a first-line TB drug, and livestock, wildlife, and zoo animals can function as TB reservoirs, infecting new people in otherwise TB-free settings. Effective control measures and integrated strategies targeting Mtb, *M. bovis*, and other TB-causing bacteria across human, animal, and environmental domains are essential to significantly reduce transmission and achieve global objectives such as the World Health Organization′s END TB Strategy. This review therefore takes a "One Health" approach, which focuses on three pillars and the connections between them: human health, animal health, and the health of their shared environment. One Health is a helpful framework for understanding complex threats like TB because it reflects the importance and interconnectedness of all three priorities, and can inspire solutions that are adaptive, interdisciplinary, and sustainable.

## Introduction

Tuberculosis (TB) is the world′s deadliest infectious disease in humans, taking 1.23 million lives and infecting 10.7 million others in 2024 [1]. The vast majority of cases are caused by *Mycobacterium tuberculosis* (Mtb), but as many as 10%–15% in low- and middle-income countries (LMICs) are attributable to *Mycobacterium bovis* [2–5]. Between 2016 and 2020, the World Health Organization (WHO) estimated that 140,000–149,000 people developed "zoonotic TB" each year, which it defined as TB in humans caused by *M. bovis* [6–10]. However, a variety of bacteria can cause TB in animals and humans. These include *Mycobacterium orygis*, *Mycobacterium caprae*, *Mycobacterium pinnipedii*, *Mycobacterium microti*, and *Mycobacterium mungi*, all of which are part of the Mtb complex (MTBC) alongside Mtb and *M. bovis* [11, 12]. High-income countries have a very low burden of *M. bovis* and zoonotic TB, constituting about 1% of patients [13, 14]. To protect humans from Mtb and *M. bovis*, they use "test-and-slaughter" in herds of livestock, pasteurize milk, conduct meat inspections, and thoroughly treat wastewater [2, 15].

**Fig. 1:**
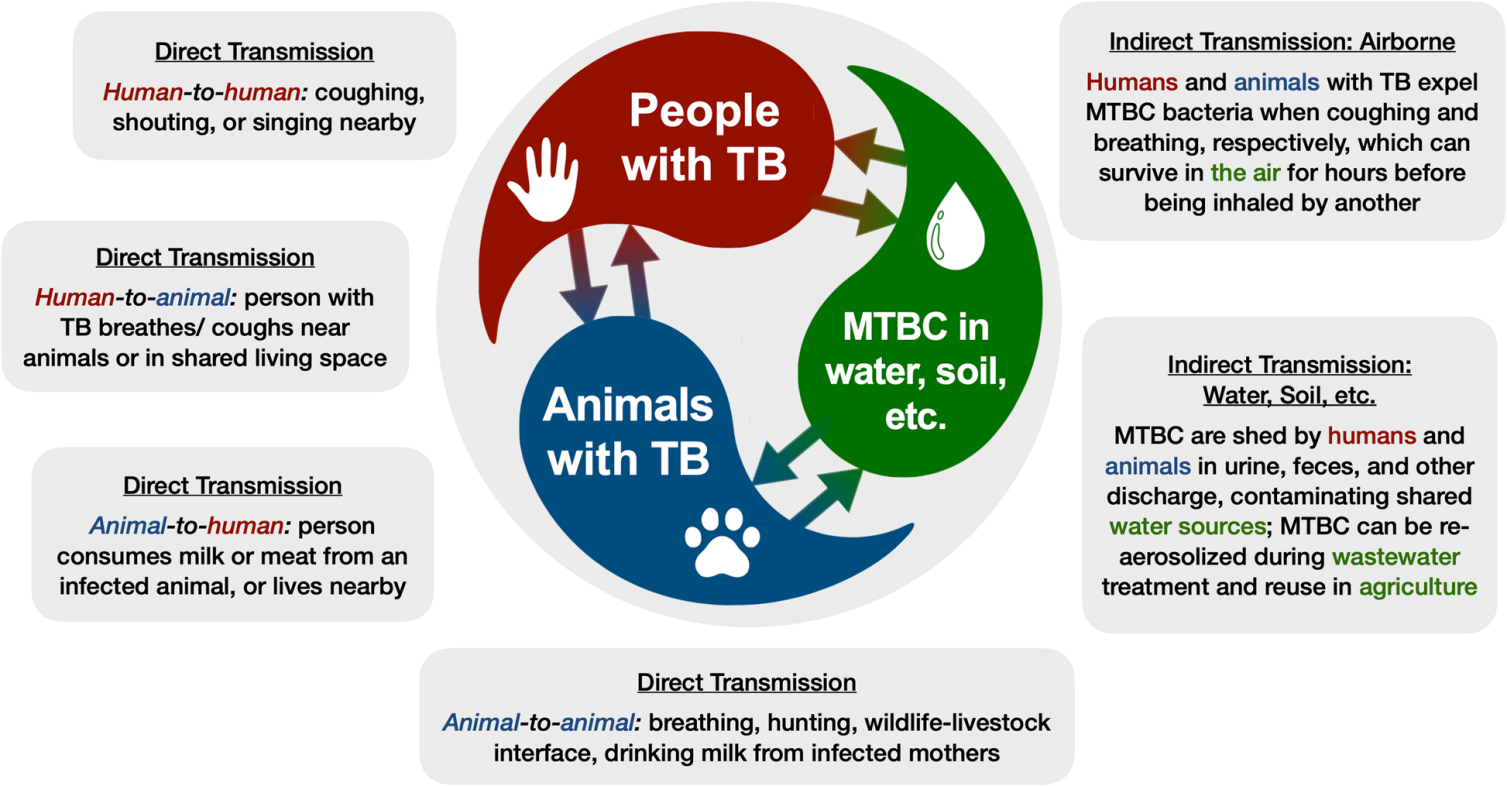
One Health model for transmission of *Mycobacterium tuberculosis*, *Mycobacterium bovis*, and other MTBC bacteria. *TB* Ttuberculosis, *MTBC* *M. tuberculosis* complex

However, these control strategies are not feasible everywhere, so over 20 million animals are estimated to have TB infection [4, 16]. Mtb, *M. bovis*, *M. orygis*, and other MTBC species can be transmitted directly and/or indirectly among humans and animals, and at times through the natural environment as well (Fig. 1) [17]. For example, elephants and cows in India and Thailand have contracted Mtb directly from their human handlers [12, 18, 19], as have a variety of zoo animals, whose infection was "associated with Mtb-infected Asian elephants in the same zoos but with no documented contact" [20]. MTBC bacteria can survive for long periods in mediums like soil, water, milk, pastures, dust, and wastewater [11, 14, 21–23], and then cause TB infection in exposed animals like guinea pigs and cattle [12, 24–26]. One Health solutions are therefore needed to ensure that MTBC bacteria are no longer spreading among humans, animals, or their shared environment.

**Fig. 2:**
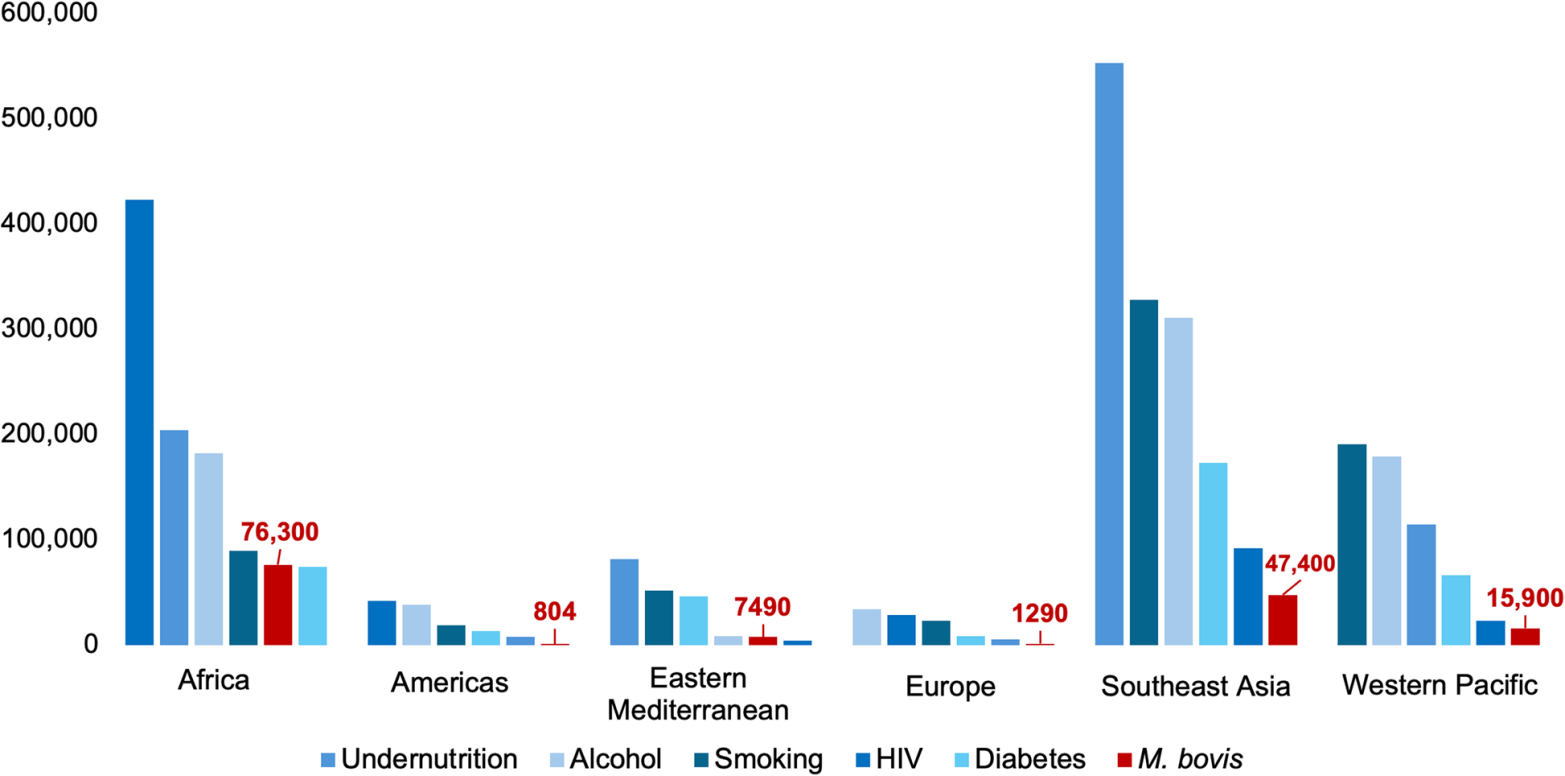
Regional estimates for annual incident cases of TB attributable to six risk factors, including *M. bovis* (by World Health Organization/Pan American Health Organization (WHO/PAHO) region). This figure presents data from two WHO sources: (1) regional estimates for *M. bovis*-attributable cases (in red) are from the WHO′s 2016 Global Tuberculosis Report [6] (Box 3.5); and (2) regional data for five risk factors (in blue) are from the online supplement to the WHO′s 2024 Global Tuberculosis Report [31] (available at https://www.who.int/teams/global-programme-on-tuberculosis-and-lung-health/data, resource titled ″TB country, regional and global profiles,″ accessed 29 April 2025). *HIV* Human immunodeficiency virus

TB in animals significantly contributes to the global TB burden in humans and poses a major challenge to TB control efforts. According to the World Organisation for Animal Health (WOAH), as many as 1 in 10 incident cases of TB globally are caused by *M. bovis* [3], or roughly 1.8 million humans in 2023. The estimate does not include other MTBC species like *M. orygis*, which is the leading cause of zoonotic TB in South Asia and has been detected on four other continents [27, 28]. The WHO′s more conservative estimate for *M. bovis* was 149,000 in 2016 [6]. Even under scrutinized estimates, *M. bovis* would rank among the top five risk factors for human TB in at least two WHO regions—exceeding the number of incident TB cases attributable to diabetes in Africa and human immunodeficiency virus infection in the Eastern Mediterranean (Fig. 2) [29]. Despite this, the WHO has not defined or described TB in animals, *M. bovis*, or zoonotic transmission in its annual Global Tuberculosis Report since the 2020 edition [10]. Therefore, health authorities should integrate *M. bovis* and the One Health approach into TB research, protocols, and programming, as continued omission may sustain transmission and jeopardize progress toward global TB reduction targets [30].

### One Health review of Mtb and *M. bovis*

#### Global incidence and prevalence

The incidence and prevalence of *M. bovis* in humans are not known because there are a variety of barriers to diagnosis and differentiation from Mtb [2]. However, researchers estimate that 10%–15% of new TB cases in LMICs are caused by *M. bovis* [3, 4], or roughly 12.1% of global cases [32]. In the five Global Tuberculosis Reports that mentioned *M. bovis* or zoonotic TB (2016–2020), the WHO concluded there were 140,000–149,000 incident TB cases and 11,400–13,400 deaths globally each year due to *M. bovis* [6]. In 2023, they collected and then published disaggregated data about MTBC detected in humans: 32 countries reported > 500 total cases of "zoonotic TB," at least 18 of which were caused by *M. orygis*, *M. caprae*, and *M. pinnipedii* (Table 1) [31]. These are extremely conservative estimates, but they demonstrate the need for broader MTBC surveillance, even in countries with low TB burdens.

**Table 1:** Incident cases of zoonotic tuberculosis reported to the World Health Organization (WHO) in 2023

Total incident cases reported		If available: disaggregation by MTBC species			
**State reporting zoonotic tuberculosis (*n* = 32)**	**Number of cases**	***Mycobacterium bovis***	***Mycobacterium orygis***	***Mycobacterium caprae***	***Mycobacterium pinnipedii***
Argentina	48	48	0	0	0
Australia	7	7	0	0	0
Austria	3	2	0	1	0
Bahrain	1	1	0	0	0
Belgium	3	3	0	0	0
Canada	30	NA	NA	NA	NA
Chile	8	8	0	0	0
Denmark	1	1	0	0	0
Ecuador	1	0	0	0	1
Fiji	3	NA	NA	NA	NA
Finland	1	1	0	0	0
Germany	50	47	0	3	0
Greece	1	1	0	0	0
Hungary	1	0	0	1	0
Ireland	8	8	0	0	0
Italy	10	10	0	0	0
Lebanon	14	14	0	0	0
Mexico	3	3	0	0	0
Netherlands	6	6	0	0	0
New Zealand	1	NA	NA	NA	NA
Norway	1	1	0	0	0
Romania	3	3	0	0	0
Spain	55	51	0	4	0
Sweden	1	1	0	0	0
Switzerland	10	10	0	0	0
Syrian Arab Republic	1	1	0	0	0
Tunisia	55	54	0	1	0
Turkey	26	25	0	1	0
United Kingdom	26	26	0	0	0
United Republic of Tanzania	30	NA	NA	NA	NA
United States of America	136	130	6	0	0
Venezuela	2	NA	NA	NA	NA

These data were published in an online supplement to the WHO′s 2024 Global Tuberculosis Report [31] (available at https://www.who.int/teams/global-programme-on-tuberculosis-and-lung-health/data, resource titled ″Zoonotic TB in 2018 and 2023,″ accessed 29 May 2026). *MTBC* *Mycobacterium tuberculosis* complex, *TB* Tuberculosis, NA stands for ″not available″ given that five states did not report disaggregated values

Data regarding the incidence and prevalence of TB in animals are even more limited. Rates vary greatly across different types of animals, regions of the world, and MTBC species. Global estimates for the prevalence of *M. bovis* range from 9% to 41% in such diverse species as cattle, buffalos, pigs, cervids, camelids, and wild boars [33, 34]. In Ethiopia, some cattle herds are "approaching 100% test positivity" for *M. bovis* [35]. In India, an estimated 21.8 million (95% CI: 16.6, 28.4) cows are infected with *M. orygis* [16], with many testing positive for multiple MTBC species [18, 23]. Other MTBC species have also been found across the animal kingdom, such as *M. caprae* in goats, deer, boars, and bison; *M. orygis* in antelopes; *M. mungi* in mongooses; *M. pinnipedii* in seals and sea lions; *M. microti* in rodents; *Mycobacterium suricattae* in meerkats; and Mtb—considered the "human strain"—in elephants, cows, chimpanzees, rhinoceroses, buffalo, boars, giraffes, and tapirs, among others [11, 19, 20, 33, 36].

#### Clinical presentation and diagnosis

In humans, TB symptoms include persistent cough, fever, weight loss, and night sweats [37]. Infection with Mtb usually leads to TB in the lungs, but people with *M. bovis* are more likely to develop extrapulmonary TB; the most common signs of *M. bovis* are cervical lymphadenopathy primarily involving tonsillar and pre-auricular lymph nodes, intestinal lesions, and chronic skin lesions (lupus vulgaris) [2]. Of the global extrapulmonary TB cases, 9%–10% are suggested to be caused by *M. bovis* [3, 4]. *M. bovis* also causes pulmonary TB, especially in reactivated cases. Pulmonary TB caused by *M. bovis* is clinically indistinguishable from that of Mtb, with similar symptoms including fever, cough, chest pain, cavitation, and hemoptysis [17].

The differentiation of TB caused by Mtb from *M. bovis* is difficult owing to their comparable clinical manifestations [2–4] and the lack of a specific point-of-care diagnostic test [14]. For example, Xpert MTB/RIF assay, which is widely used in the detection of human TB, cannot differentiate Mtb from *M. bovis* [38]. *M. bovis* shows dysgonic growth pattern—which is slower and sparser than the growth of Mtb—in solid or liquid culture media such as Lowenstein-Jensen and Mycobacteria Growth Indicator Tube, accentuated by the presence of glycerol in the media that facilitates Mtb but inhibits the growth of *M. bovis* [39]. While use of pyruvate enables growth of *M. bovis* in the culture system, with crude phenotypic differentiation possible from colony characteristics, further testing such as polymerase chain reaction (PCR) test or DNA sequencing is needed for confirmatory diagnosis, as both Mtb and *M. bovis* grow in pyruvate-based media. These technological shortcomings are compounded by the lack of specimens in extrapulmonary TB, for which *M. bovis* has predilection [3, 40]. In the absence of more accessible and cost-efficient diagnostic tools, misdiagnosis is inevitable. Unlike Mtb, strains of *M. bovis* are naturally resistant to the first-line TB treatment drug pyrazinamide [41, 42], increasing the likelihood of treatment failure and catastrophic outcomes including prolonged suffering, permanent disability, death, and financial crisis [4, 43, 44].

Much like humans, animals with MTBC infection may present with weight loss and lung lesions [45, 46]. However, often, animals with TB do not cough [46] and have clinical lesions situated deep in their respiratory and digestive tracts [33, 47, 48]. Symptoms and signs such as weakness, shortness of breath, and enlarged lymph nodes can be hard to notice [17]. The unreliability of existing diagnostic tools such as tuberculin skin test in vaccinated animals, and in animals in contaminated environments, adds to the challenge [4, 14, 18, 33, 49, 50]. Some wildlife can only be diagnosed with species-specific culture reagents [45]. As such, animals with MTBC infection are usually only diagnosed postmortem [11, 14].

MTBC species like *M. bovis* can be identified and differentiated using specific diagnostic tools. PCR assays, region of difference, spacer oligonucleotide typing (spoligotyping), variable number of tandem repeats typing of mycobacterial interspersed repetitive units, restriction fragment length polymorphism, and whole genome sequencing (WGS) can specifically detect *M. bovis* [36, 51–53]. By assessing strain relatedness, WGS can also reveal *M. bovis* transmission patterns, with important public health implications [54–56]. Unfortunately, the above tests are all currently limited by their need for advanced technology and laboratory capacity, precluding their use in decentralized or programmatic settings [14, 56, 57].

#### Direct transmission routes

TB transmission usually happens when MTBC bacteria are carried through the air by droplets—often expelled by a cough—and then inhaled by another person or animal. Direct airborne transmission of Mtb is especially common when people share their homes with livestock [2, 3, 14] or live in crowded settings like monasteries [58]. *M. bovis*, by contrast, has only been observed spreading among people who have immunodeficiency disorders [39, 47]; human infections with *M. bovis* are almost always traced to TB-positive livestock and pets, or the ingestion of contaminated milk or meat [4, 14, 41]. Zoonotic TB—encompassing both species—is especially common among people who consume raw, unpasteurized, or undercooked animal products [4, 15, 47], and those with occupational exposures to animals, like veterinarians, smallholder farmers, zookeepers, meat inspectors, and butchers [2, 3, 14, 19].

MTBC infection can be transmitted directly among animals, as well as back-and-forth with humans, through "aerogenous, digestive, transplacental, and cutaneous" routes [47]. For example, newborn calves have been infected with MTBC by drinking their mothers′ milk [18, 49], brushtail possums by licking one another [11], and domestic cats by hunting voles [12]. Some wildlife can serve as TB reservoirs; species like the European badger (*Meles meles*) and African buffalo (*Syncerus caffer*) can live with *M. bovis* infection for years, transmitting it to new animals and contaminating the environment [59, 60]. In addition to reservoir hosts, MTBC have "spillover hosts" such as feral pigs (*Sus scrofa*) spreading it to other species [61]. Depending on the ecosystem and type of MTBC, animals like red deer (*Cervus elaphus*) can be reservoir hosts in some regions but spillover hosts in others [62, 63]. These transmission dynamics are relevant to human TB control because they can involve animals in proximity to people, like domesticated cows and zoo animals [11, 19, 41]. This risk of "spill-over and spill-back between species" underscores the importance of a One Health approach because eliminating TB in only humans or animals will not be enough to end the global epidemic [11, 20, 27] (Fig. 1).

#### Indirect transmission routes

Indirect transmission among humans and animals is often facilitated by the environment, necessitating a One Health approach. Researchers have suggested that MTBC bacteria can be aerosolized (and then inhaled) by: surgeries, autopsies, and other medical procedures on TB patients; sewage treatment; reuse of wastewater for irrigation; and when elephants breathe in deeply through their trunks to "investigate their environment" [15, 20, 24, 26, 64]. Elevated dew point temperatures, projected to increase this century [65], may enhance MTBC survival in the air, thereby facilitating progression to active disease following host infection [11, 66, 67].

Although TB is considered an airborne disease, MTBC bacteria can also spread through solids and liquids, contaminating pastures, watering holes, food, and other surfaces [11, 14, 23, 24]. This is because animals shed *M. bovis* in their breath, mucus, sputum, urine, feces, milk, and wound discharges [2, 11, 23, 26, 46], and humans can shed Mtb in their urine and feces [20, 22, 24]. Bacteria that settle into the water or soil can then stay virulent and re-culturable for 3–6 months [18, 22, 66]. MTBC can be isolated from carpets, dust, and wood for 18–88 days after contamination [22, 24]. The occurrence of indirect transmission is supported by the identification of identical TB strains in animals and humans with no apparent direct contact or respiratory interaction [12, 14, 19, 20, 25, 46], as well as in water and soil samples from the national parks [23], wild habitats [68], and sewage raceways [22, 26, 64] around them. Animals are more likely than humans to contract TB indirectly because they engage more closely with the natural environment, further emphasizing the need for One Health solutions [12] (Fig. 1).

It is important to acknowledge that the presence of MTBC in the environment does not invariably result in TB risk or infection, as both humans and animals may be exposed without adverse effects [24, 69]. However, evidence indicates that animals can contract infections through water, dust, and food [12, 18, 20, 26, 48]. Although the potential for humans to acquire TB infection from environmental sources remains unproven, there are reports of individuals with no other TB risk factors developing the disease following exposure to contaminated medical waste [20], and those with prolonged exposure to sewage work exhibiting relatively higher TB rates [70]. Even if MTBC bacteria in the environment do not pose a direct transmission threat, whole genome sequencing and analysis of MTBC samples can assist experts in identifying the sources and pathways of TB transmission [24, 69].

### Control strategies for *M. bovis*

#### Animals: "test-and-slaughter" vs. "test-and-segregate"

Instead of focusing on prevention, care, or cures for affected animals, high-income settings like the US and UK largely rely on "test-and-slaughter" to eliminate *M. bovis* in herds; to prevent transmission to humans, they also have expensive systems for pasteurization, meat inspection, and high-risk worker protections [13, 15, 38]. According to the WOAH, "test-and-slaughter" has been used successfully in these areas for over 100 years "because it definitively removes the source of infection from the population and eliminates the opportunity for further spread of MTBC species," particularly when participants are compensated [71]. Providing anti-TB drugs to livestock rather than slaughtering them is explicitly not recommended by the WHO and WOAH, and "TB drugs... cannot be used in food-producing animals" under European Union regulations, because animals undergoing TB treatment can still transmit it and contribute to antimicrobial resistance [2, 71–73]. Other experts discourage treatment because human regimens like chemotherapy are not as effective in animals or against extrapulmonary TB, which *M. bovis* more often causes [4, 39, 40, 47].

There are a variety of reasons why "test-and-slaughter" is not acceptable or effective in other settings. The practice faces ethical opposition when applied to species that are endangered or hold profound cultural and religious importance [11, 18, 42, 45]. Particularly in low-resource settings, it can be financially and logistically infeasible for people to routinely slaughter and replace their livestock without compensation [14, 38, 71]. Further, test-and-slaughter can disincentivize TB surveillance; if every positive test result leads to the culling of an animal, people may choose not to test them at all [74].

Fortunately, there are alternative ways to prevent veterinary TB. Immunizing cows with the Bacillus Calmette-Guérin (BCG) vaccine seems to reduce disease severity and transmission [35, 50, 74], particularly when combined with other vaccines [18]. European badgers can also gain some immune protection from BCG via oral baits [12]. Since 2018, Ireland has largely transitioned from killing wild badgers to vaccinating them; an early pilot showed that vaccination was "not inferior to targeted badger-culling" as a way to limit TB incidence among cows in their shared environment [75, 76]. In cattle, primary prevention is possible through "early calf segregation" or "test-and-segregate," whereby newborn calves are quickly separated from their TB-positive mothers, protecting them from the MTBC in their breath and milk [18, 49, 74]. On intensive dairy farms in Ethiopia, test-and-segregate is reportedly more efficient (12 vs. 18 months) and cost-effective (US$52 vs. US$107 per animal) than test-and-slaughter to achieving TB-free herds [77]. Other studies have found that test-and-segregate can be even more effective when combined with BCG vaccines [49]. Although there is no precedent for regional- or national-level BCG immunization in animals, it represents an accessible point of intervention with few downsides or risks.

#### Humans: policy and pyrazinamide

High-income settings have demonstrated that it is possible to prevent *M. bovis* infection in humans, but it requires a multi-pronged, cost intensive One Health approach [15]. Key components include cross-border TB surveillance programs for humans and animals, pasteurization mandates, occupational health policy improvements, inter-species vaccine research, wastewater surveillance, and enhanced sewage treatment [13, 18, 23, 39, 64]. But these can be cost-prohibitive, culturally incompatible, or unrealistic to enforce in low-resource settings, particularly in the climate crisis, with unpredictable changes to ecological systems, migration, and interface between species [11, 14, 41].

Further, the spread of *M. bovis*, which is universally resistant to pyrazinamide (PZA) [53], could accelerate the emergence of multidrug-resistant TB (MDR-TB), which is associated with higher mortality, treatment failure, and cost. MDR-TB is projected to cause 75 million deaths between 2015 and 2050 [78], posing a significant global health security threat. In 2023 alone, approximately 400,000 individuals developed MDR-TB [31]. These cases were assumed to be Mtb infections, but many may have actually been *M. bovis* [44, 57, 79–82]. These rifampin-resistant or MDR-TB estimates were calculated based on TB detection by Xpert MTB/RIF or the solid and liquid TB culture systems, but these systems cannot differentiate Mtb from *M. bovis*. The *rpoB* gene targeted by Xpert MTB/RIF assay is present in Mtb and other members of MTBC, including *M. bovis*. Moreover, a systematic review in 2023 estimated the global prevalence of pyrazinamide resistance to be 57% (95% CI: 48%–65%) in people with MDR-TB and 29% (95% CI: 23%–35%) in all TB [83]. *M. bovis* strains are intrinsically resistant to PZA due to naturally selected mutations in *pncA* gene [53, 84]. Approximately 25%–50% of the PZA mono-resistant cases were shown to be due to *M. bovis* in retrospective studies from Germany and the US [80, 81, 85]. On the other hand, PZA resistance has also been linked to previous exposure to anti-TB drugs, suggesting acquired resistance in Mtb [79, 80, 86, 87]. On the whole, it is reasonable to summarize that at least some of the globally observed phenotypic resistance to PZA may be attributable to *M. bovis*. Programmatically, the recommendation by the WHO for universal drug susceptibility testing for new TB is a step in the right direction [31]. Since many PZA-resistant cases may be caused by *M. bovis* [5, 84, 88–90], TB disease resistant to PZA can be additionally considered for *M. bovis* testing [15, 44, 81].

#### Both: One Health research and solutions

The field of veterinary TB is relatively sparse and decentralized, since it connects such diverse sectors as public health, food safety, agriculture, environmental biology, animal welfare, and clinical medicine. Perhaps as a result, many different terms have been used to describe the same threats and processes, preventing knowledge-sharing and cross-collaboration. But high-risk groups of all species deserve to benefit from existing knowledge about TB, and going forward, researchers can design mutually beneficial One Health studies, like testing or comparing TB vaccines, diagnostic tools, and treatments in humans vs. animals [15, 40, 46, 50].

Crucially, basic terminology requires standardization. In key guidelines and recommendations, the WHO, WOAH, and other international agencies distinguish zoonotic TB from bovine TB but limit the scope of both definitions to TB caused by *M. bovis* [27, 71, 91]. For example, in their joint "Roadmap for Zoonotic Tuberculosis," ′zoonotic TB′ refers to disease caused by *M. bovis* infection in people and ′bovine TB′ refers to disease caused by *M. bovis* infection in animals" [91]. Such binary terms imply there are differences between the diseases, their targets, or sources of infection, even though MTBC species can be practically indistinguishable and cycle bidirectionally between humans and animals [2, 3, 14]. Humans are vulnerable to *M. bovis*, but health authorities seldom treat animal exposure as a TB risk factor [3]; conversely, animals are susceptible to Mtb, but there is a lack of consensus regarding appropriate treatment [2, 19]. Definitions should also encompass other MTBC species contributing to TB burden in humans and animals, such as *M. orygis* [27, 28, 92]. Researchers have therefore proposed defining zoonotic TB as "a form of human tuberculosis acquired from an animal source... diagnosed based on epidemiological evidence of relevant animal or environmental exposure, and/or microbiological confirmation of a [MTBC] subspecies usually found in animals" [27]. The term "veterinary TB" can be used to describe MTBC infection or disease in animals, encompassing more mycobacterium species, non-bovine hosts, and transmission from both humans and fellow animals [93, 94].

In addition, to advance global goals like the END TB Strategy, animals and zoonosis must re-enter global health discourse about TB—particularly by authorities like the WHO, which has not described them in its annual TB report since the 2020 edition [10]. National TB programs should alert people to the threat of zoonotic infection in their guidelines and protocols [3], and create information campaigns tailored to high-risk groups, like people who drink unpasteurized milk [18, 95] or have frequent occupational exposures, such as elephant handlers, butchers, and sewage workers [3, 19, 26]. When possible, human and animal health authorities should exchange TB case reports and collaborate on research [23, 39, 42]. A One Health approach is essential for addressing complex threats such as *M. bovis*, as compartmentalized research is currently impeding progress against the disease [2, 3, 11, 14, 24].

## Conclusions

The risks of Mtb, *M. bovis*, and other MTBC species are significant and implicate animals, people, and the natural environment (Fig. 1). Even when human TB rates are negligible, animal and environmental reservoirs can be public health hazards [27, 41]. The elimination of TB and achievement of global goals may be hindered if a One Health approach is not widely adopted [20, 44]. In addition, accounting for more MTBC species in human clinical research and guidelines—as well as in veterinary research and guidelines—could help mitigate TB’s transmission, drug resistance, and morbidity [14, 42, 91].

In conclusion, TB is not only a threat to people around the world, but also to animals, including livestock, wildlife, pets, and zoo animals. If TB surveillance, research, and treatments continue to focus exclusively on humans, then the evidence base will provide an incomplete picture of the TB landscape and may limit progress against it. Zoonoses like TB challenge the boundaries of animal and human science and medicine, so One Health is an excellent if not necessary approach to eliminating them [38, 39].

## Data Availability

Data sharing is not applicable to this article as no datasets were generated or analyzed during the current study.

## Abbreviations

BCG: Bacillus Calmette-Guérin (vaccine)
LMICs: Low- and middle-income countries
MDR-TB: Multidrugresistant-tuberculosis
Mtb: *Mycobacterium tuberculosis*
MTBC: *Mycobacterium tuberculosis* complex
*M. bovis*: *Mycobacterium bovis*
*M. caprae*: *Mycobacterium caprae*
*M. microti*: *Mycobacterium microti*
*M.** mungi*: *Mycobacterium* *mungi*
*M. orygis*: *Mycobacterium orygis*
*M. pinnipedii*: *Mycobacterium pinnipedii*
*M. suricattae*: *Mycobacterium suricattae*
PCR: Polymerase chain reaction (assay/test)
PZA: Pyrazinamide
RR: Rifampin-resistant
TB: Tuberculosis
WGS: Whole genome sequencing
WHO: World Health Organization
WOAH: World Organisation for Animal Health
